# The Melatonin Signaling Pathway in a Long-Term Memory In Vitro Study

**DOI:** 10.3390/molecules23040737

**Published:** 2018-03-23

**Authors:** Jin-Young Sung, Ji-Hyun Bae, Jong-Ha Lee, Yoon-Nyun Kim, Dae-Kwang Kim

**Affiliations:** 1 Department of Medical Genetics, Hanvit Institutute for Medical Genetics, School of Medicine, Keimyung University, Daegu 42601, Korea; jysunny486@hanmail.net (J.-Y.S.); bjh84@dsmc.or.kr (J.-H.B.); 2 Department of Biomedical Engineering, School of Medicine, Keimyung University, Daegu 42601, Korea; segeberg@kmu.ac.kr; 3 Dongsan Medical Center, Department of Internal Medicine, Keimyung University, Daegu 42931, Korea; ynkim@dsmc.or.kr

**Keywords:** melatonin, long-term memory, CREB signal, cellular senescence

## Abstract

The activation of cyclic adenosine monophosphate (cAMP) response element-binding protein (CREB) via phosphorylation in the hippocampus is an important signaling mechanism for enhancing memory processing. Although melatonin is known to increase CREB expression in various animal models, the signaling mechanism between melatonin and CREB has been unknown in vitro. Thus, we confirmed the signaling pathway between the melatonin receptor 1 (MT1) and CREB using melatonin in HT-22 cells. Melatonin increased MT1 and gradually induced signals associated with long-term memory processing through phosphorylation of Raf, ERK, p90RSK, CREB, and BDNF expression. We also confirmed that the calcium, JNK, and AKT pathways were not involved in this signaling pathway by melatonin in HT-22 cells. Furthermore, we investigated whether melatonin regulated the expressions of CREB-BDNF associated with long-term memory processing in aged HT-22 cells. In conclusion, melatonin mediated the MT1-ERK-p90RSK-CREB-BDNF signaling pathway in the in vitro long-term memory processing model and increased the levels of p-CREB and BDNF expression in melatonin-treated cells compared to untreated HT-22 cells in the cellular aged state. Therefore, this paper suggests that melatonin induces CREB signaling pathways associated with long-term memory processing in vitro.

## 1. Introduction

Melatonin is the main hormone produced by the pineal gland [1] and decreases with age in humans [2,3]. Melatonin also phosphorylates CREB, which is correlated with enhancements in learning and memory in mice and rats [4,5]. The phosphorylation of CREB by melatonin is likely mediated through the melatonin receptors, melatonin receptor 1 (MT1) and melatonin receptor 2 (MT2), according to a study of the nervous system [6]. In addition, the MT1/MT2 receptors are present in the cell membrane; there are other intracellular binding sites for melatonin as well [7]. Also, melatonin receptors have been found in the hippocampus of various animals [8,9,10]. These studies suggest a role for melatonin, acting via specific receptors in the hippocampus cells. In addition, MT1 and MT2 receptors are widely expressed in the hippocampus, since Musshoff and colleagues found a detectable expression of transcripts in the isolated hippocampal subregions dentate gyrus, CA3, CA1, and subiculum [11]. Here also, there are non-receptor mediated actions of melatonin that protect the pyramidal cells of the hippocampus and long-term potentiation (LTP) from damage. Some of the protective action of melatonin may be receptor-independent via free radical generating mechanisms [12]. This protection also relates to learning and memory [13].

Melatonin is associated with a variety of signaling mechanisms including the ERK and MAPK pathways [14]. However, the intermediate pathways that regulate interactions between melatonin receptors and p-CREB have yet to be fully described in the hippocampus. An understanding of melatonin’s cellular actions will provide valuable insight into the biological mechanisms of memory. In addition, the levels of melatonin vary throughout animal and human life and are known to decrease with age [15]. Though there may be some exceptions to this general rule, the reduced levels of the melatonin in aged individuals have been proposed as one of the important factors in the development of age-related neurodegenerative disorders [16]. To date, melatonin administration shows memory enhancement effects in several memory– and age–related animal models, including TS65DN mice (a model of Down’s syndrome) [17] and senescence-prone mouse-8 (SAMP8) mice [18,19]. Moreover, melatonin attenuates memory impairment in the d-galactose-induced aging model [20,21]. Because the effects of melatonin and CREB signaling pathway on memory processing are not well known, it is critical to investigate the potential mechanisms of melatonin. According to previous references, it has been revealed that melatonin is involved in the signal of memory formation in vivo, but the mechanism of memory formation in vitro and in vivo is not clear. Therefore, we want to reveal the signaling pathway associated with long-term memory processing in an in vitro study.

CREB is activated by phosphorylation (p-CREB) and acts as a transcription factor to increase the expression of brain-derived neurotrophic factor (BDNF), which can enhance hippocampal memory processing [22]. It is also a component of intracellular signaling events that regulate the circadian rhythms of memory [23], long-term memory [24], and a variety of downstream effectors in the hippocampus [25]. In addition, CREB signaling is activated through several pathways that are complemented by the cAMP/PKA signaling pathway in mouse fibroblast cells [26]. However, the levels of CREB expression gradually decrease with age in the hippocampus and prefrontal cortex [27] and can contribute to cognitive deficits [25].

Immortalized cell lines have been used as the most commonly model for relevant mechanistic and pharmaceutical studies in vitro. With particular concerns for memory, hippocampal neuronal cell lines are very limited, of which HT-22 cells appear to be one of the most commonly used [28]. HT-22 cells are a sub-cloned cell line from HT-4 cells, which are immortalized mouse hippocampal neuronal cells [29]. For this reason, HT-22 cells have been used as a hippocampal neuronal cell model in numerous studies [30,31]. This cell line also provides a model system to study many neurodegenerative disorders such as Alzheimer’s and Parkinson’s disease.

In the present work, we first confirmed the long-term memory signal in the mechanism of melatonin-mediated CREB-BDNF in HT-22 cells. In conclusion, melatonin increased the expression of MT1, activated phosphorylation of Raf, ERK, p90RSK, and CREB, and increased the expression of BDNF. Also, melatonin significantly increased the levels of p-CREB and BDNF expression compared to no melatonin treatment in aged HT-22 cells in vitro. These results, which pertain to the melatonin–CREB signaling pathway, may provide therapeutic treatment strategies for age-related cognitive deficits and neurodegenerative disorders.

## 2. Results

### 2.1. Melatonin Induces the p-CREB and BDNF Associated with Long-Term Memory Processing and Appears to Be Related to the Difference in Efficacy of Melatonin Receptor Agonists between HT-22 Cells and H19-7 Cells

**Figure 1:**
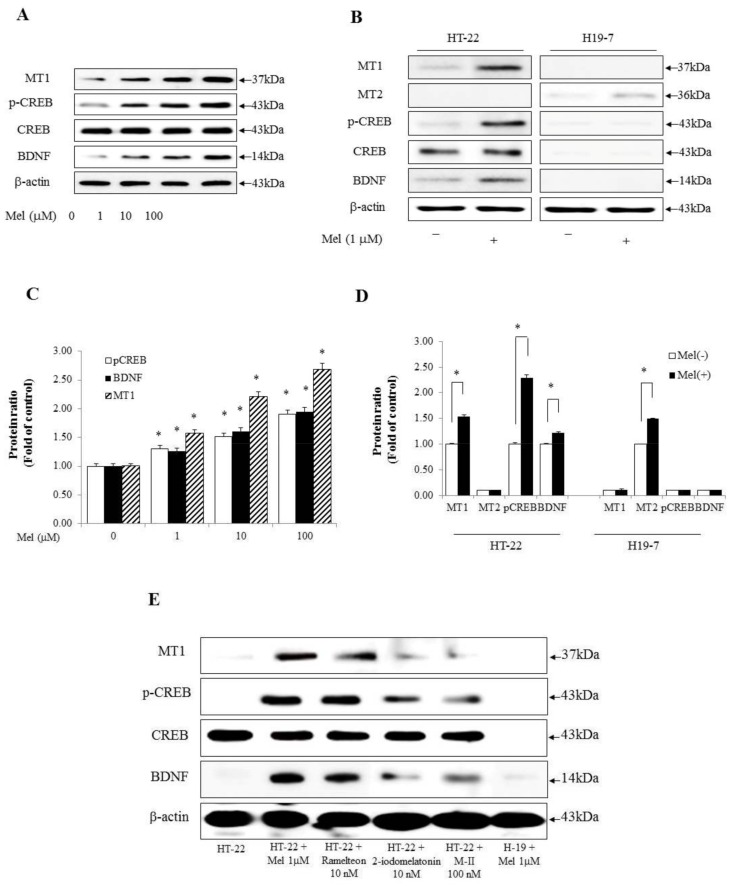

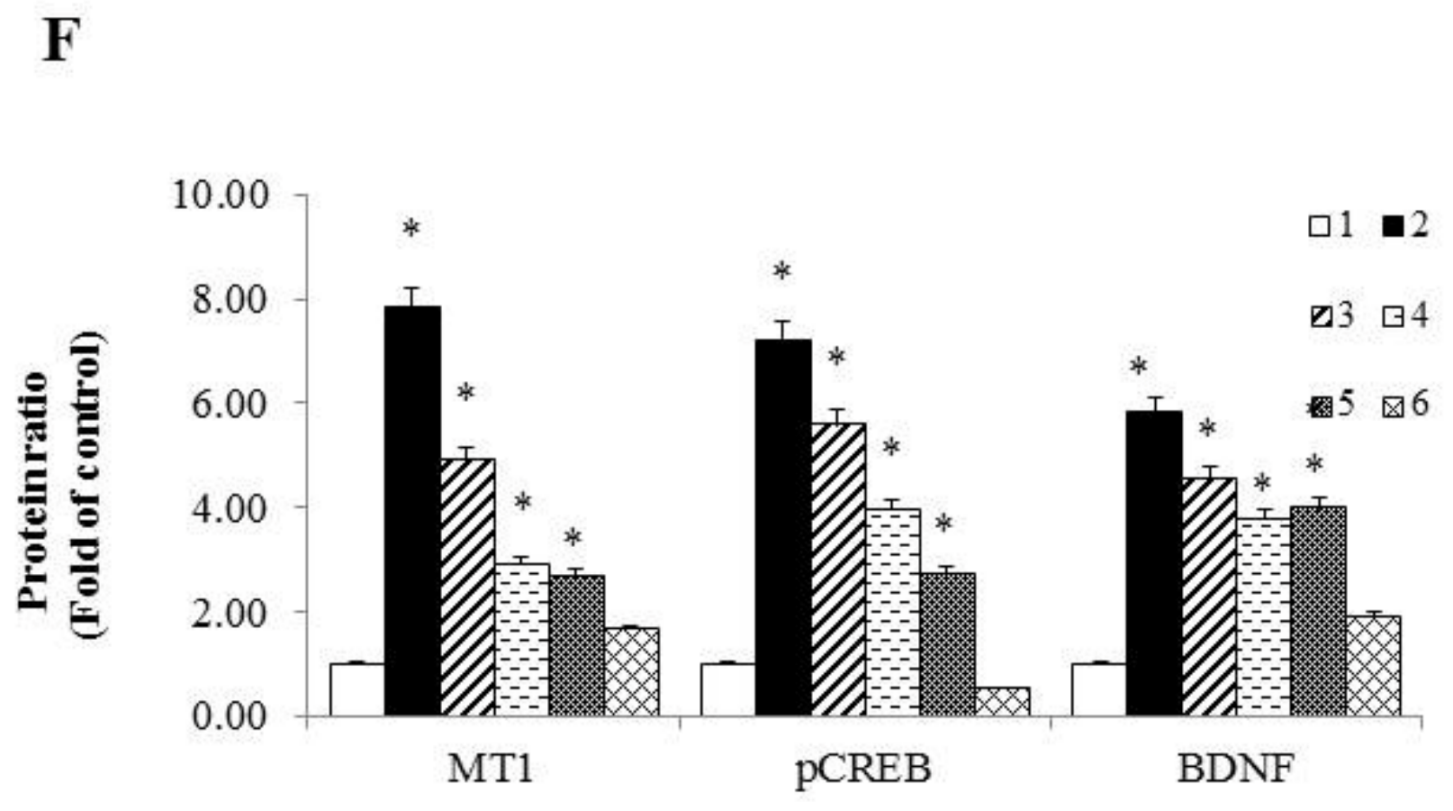
Melatonin appeared to account for the difference in expression of melatonin receptors and kinases associated with memory processing between HT-22 cells and H19-7 cells. (**A**) HT-22 cells were treated with melatonin (0, 1, 10, or 100 µM) for 2 h and the expression of MT1, p-CREB, CREB, and BDNF was shown by Western blot analysis; (**B**) Western blot analysis of MT1, MT2, p-CREB, CREB, and BDNF in two cell lines. HT-22 and H19-7 cells were treated with 1 µM melatonin for 2 h; (**C**) A graph represented the densitometry analysis of Figure 1A. * *p* < 0.05 versus each control group; (**D**) A graph representing the densitometry analysis of Figure 1B. * *p* < 0.05 versus each absence of melatonin; (**E**) Western blot analysis of MT1, p-CREB, CREB, and BDNF with melatonin agonists in two cell lines. HT-22 and H19-7 cells were treated with 1 µM melatonin, 100 nM ramelteon metabolite M-II (M-II), 10 nM 2-iodomelatonin, and 10 nM ramelteon for 2 h; (**F**) A graph representing the densitometry analysis of Figure 1E (Respectively, 1. HT-22, 2. HT-22 + Mel 1 µM, 3. HT-22 + Ramelteon 10 nM, 4. HT-22 + 2-iodomelatonin 10 nM, 5. HT-22 + ramelteon metabolite M-II (M-II) 100 nM, 6. H-19 + Mel 1 µM). * *p* < 0.05 versus melatonin non- treatment in HT-22. All experimental values were given as means ± S.E.M (*n* = 3).

We determined that MT1, p-CREB, and BDNF may be promoted in a dose-dependent manner through melatonin in hippocampal cells. HT-22 cells were incubated in the presence of various concentrations of melatonin (0, 1, 10, or 100 μM) for 2 h. The results showed that 0, 1, 10, and 100 μM melatonin treatments increased MT1, p-CREB, and BDNF in a dose-dependent manner (Figure 1A,C). HT-22 cells are immortalized mouse hippocampal neuronal cells, while H19-7 cells are generated from rat hippocampal neuronal cells. Initially, we tested two cell lines to investigate the effects of melatonin. Melatonin treatment showed the difference in the expression of melatonin receptors and kinases associated with memory processing between the two cell lines. As shown in Figure 1B,D, the MT1 receptor was significantly increased after melatonin treatment, whereas the MT2 receptor was not detected in HT-22 cells. In addition, the levels of p-CREB and BDNF were increased by melatonin in HT-22 cells. However, MT1 receptor, p-CREB, and BDNF were not detected in H19-7 cells. Instead, MT2 receptor appeared in H19-7 cells. Thus, for the next round of the experiments, we selected HT-22 cells. Some studies have reported high-affinity melatonin binding sites in the hippocampal region [32,33]. Therefore, we again confirmed the experiment with various melatonin agonists to determine whether melatonin mediates its effect on MT1 receptors in HT-22 cells. To identify differences in the level of expression of the MT1 receptor, p-CREB, and BDNF, we used ramelteon, 2-iodomelatonin, and ramelteon metabolite M-II as melatonin receptor agonists in HT-22 and H19-7 cells [34]. We found that melatonin alone strongly increased the levels of MT1 receptor, p-CREB, and BDNF in HT-22 cells than ramelteon, 2-iodomelatonin, and ramelteon metabolite M-II (Figure 1E,F). Collectively, these data demonstrate that the expression of MT1-CREB-BDNF was mediated by melatonin in HT-22 cells.

### 2.2. Melatonin May Be Related to the CREB Signaling Pathway in HT-22 Cells

**Figure 2:**
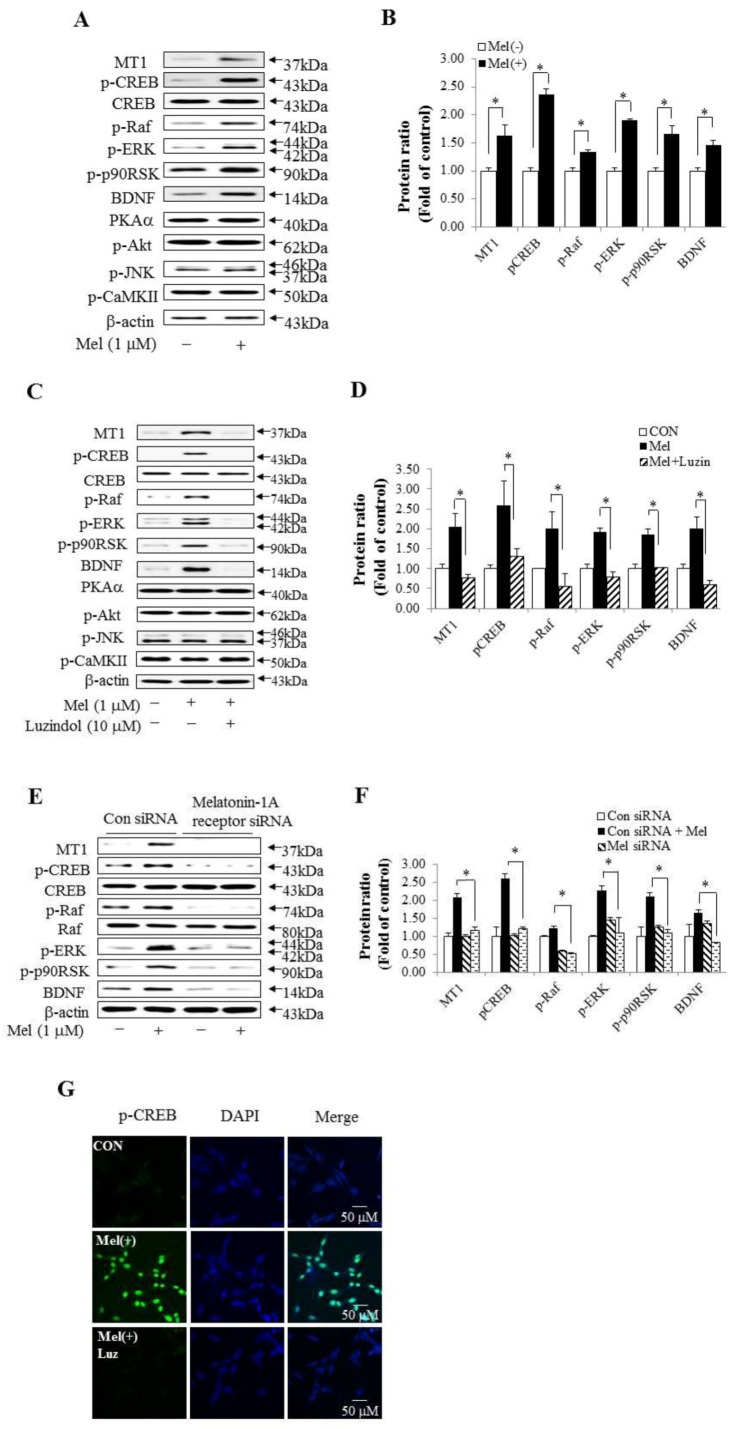
Melatonin signaling was related with the CREB signaling pathway and associated with long-term memory processing in HT-22 cells. (**A**) Melatonin regulated the expression of kinases associated with long-term memory processing. HT-22 cells were treated with 1 µM melatonin for 2 h. The expressions of MT1, p-CREB, CREB, p-Raf, Raf, p-ERK, p-p90RSK, BDNF, PKAα, p-AKT, p-JNK, and p-CaMKII were confirmed by Western blot analysis; (**B**) A graph representing the densitometry analysis of Figure 2A. * *p* < 0.05 versus each absence of melatonin. Pharmacological inhibitor and genetic inhibition of melatonin regulated the level of proteins associated with the CREB signaling pathway; (**C**) Luzindole (10 µM, 1 h) abolished the activity of the melatonin-induced CREB signaling pathway in HT-22 cells; (**D**) a graph representing the densitometry analysis of Figure 2C. * *p* < 0.05 versus treatment of melatonin alone; (**E**) HT-22 cells were transfected with control siRNA and melatonin-related receptor siRNA for 48 h, and then stimulated with melatonin. The expression of MT1 and CREB signaling pathway associated with long-term memory processing was inhibited by melatonin-1A receptor siRNA despite treating melatonin; (**F**) A graph representing the densitometry analysis of Figure 2E. * *p* < 0.05 versus control siRNA group + melatonin treatment. All experimental values were given as means ± S.E.M (*n* = 3); (**G**) Localization of p-CREB was regulated by melatonin in HT-22 cells. Representative photomicrographs of p-CREB immunofluorescent staining (green) to show the distribution of green staining cells treated with 10 µM luzindole for 1 h and then treated with melatonin. The cell nuclei were stained with DAPI (blue). Photomicrographs were taken at the same magnification. (Scale bar = 50 µM).

Previous studies reported that melatonin led to the phosphorylation of CREB [35]. First, we examined whether melatonin regulated protein levels associated with the upstream and downstream of CREB in HT-22 cells. The levels of p-Raf, p-ERK, p-p90RSK, and BDNF proteins were increased after melatonin treatment compared with each control (Figure 2A,B). However, PKAα, p-AKT, p-JNK, and p-CaMKII protein levels did not change after melatonin treatment. Second, we determined whether inhibition of melatonin could reverse protein levels associated with upstream and downstream of p-CREB in HT-22 cells. Luzindole was used as a nonselective competitive melatonin receptor antagonist [36]. We administered 10 μM luzindol to HT-22 cells and then cultured the cells supplemented with 1 μM melatonin. The MT1 receptor, p-CREB, p-Raf, p-ERK, p-p90RSK, and BDNF expression was increased in HT-22 cells treated with melatonin compared in untreated HT-22 cells, and these proteins appeared at the same level in HT-22 cells treated with luzindole and melatonin and HT-22 cells not treated with melatonin. However, luzindole did not influence PKAα, p-AKT, p-JNK, or p-CaMKII expression (Figure 2C). Next, to confirm the importance of melatonin in this pathway, we tested the effects of RNA interference on the MT1 receptor pathway. Melatonin-1A receptor siRNA significantly reduced MT1 receptor expression and Raf-ERK-p90RSK-dependent p-CREB and BDNF expression were also reduced (Figure 2E,F). In addition, to determine whether p-CREB protein is localized to the nucleus by melatonin in HT-22 cells, we performed an immunofluorescence analysis. Representative photomicrographs of immunofluorescent staining (green) showed the distribution of p-CREB in cells treated with melatonin alone, whereas the co-treatment of melatonin and luzindole did not show p-CREB (Figure 2G). The cell nuclei were stained with DAPI (blue) and the co-localization of p-CREB and nuclei was marked in yellow-green. These results showed that localization of p-CREB was regulated by melatonin in HT-22 cells.

### 2.3. Other Signaling Pathways, Except for the Raf–ERK Pathway, Did Not Affect the Expression of pCREB through Melatonin

**Figure 3:**
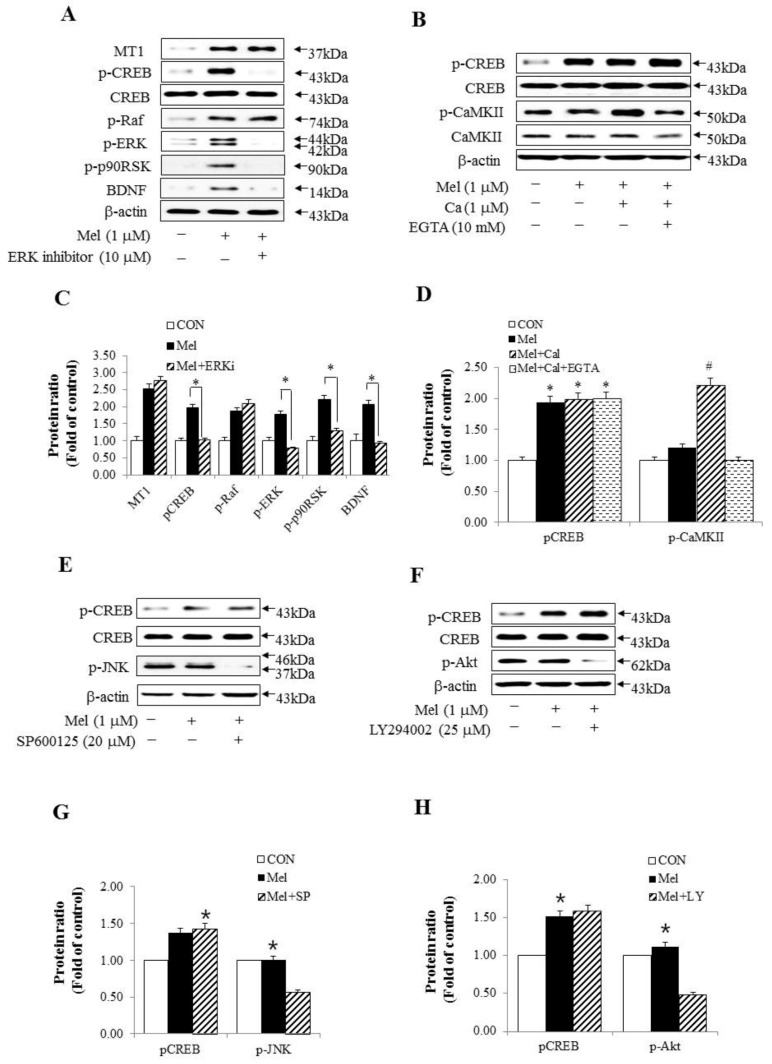
Three other signals apart from the Raf-ERK pathway did not influence the levels of p-CREB by melatonin. (**A**) ERK inhibitor (10 µM, 1 h) abolished p-ERK, p-p90RSK, and BDNF along with p-CREB in HT-22 cells. However, the ERK inhibitor did not influence the levels of MT1 and p-Raf; (**B**) Effects of calcium (1 µM, given 40 min after melatonin treatment) and chelator EGTA (10 mM, 2 h) had nothing to do with the levels of p-CREB induced by melatonin; (**C**) a graph representing the densitometry analysis of Figure 3A. * *p* < 0.05 versus treatment of melatonin alone; (**D**) A graph representing the densitometry analysis of Figure 3B. * *p* < 0.05 versus control in p-CREB group and # *p* < 0.05 versus control in p-CaMKII group; (**E**) JNK inhibitor (SP600125, 20 µM, 1 h) did not influence the expression of p-CREB despite melatonin treatment in HT-22 cells; (**F**) Akt inhibitor (LY294002, 25 µM, 1 h) did not affect the expression of p-CREB despite melatonin treatment in HT-22 cells; (**G**) a graph representing the densitometry analysis of Figure 3E. * *p* < 0.05 versus each control group; (**H**) A graph representing the densitometry analysis of Figure 3F. * *p* < 0.05 versus each control group. All experimental values are given as means ± S.E.M (*n* = 3).

We hypothesized that melatonin, through the MT1–Raf–ERK–p90RSK–CREB signaling pathway, may be a major signaling component of the in vitro memory processing pathway in HT-22 cells. To examine the molecular mechanisms of melatonin, we studied upstream and downstream mediators of the ERK pathway using p-CREB expression. ERK inhibition by FR180204 [37] with melatonin treatment specifically reduced p-CREB expression but did not inhibit the phosphorylation of Raf and MT1 receptor expression (Figure 3A,C). We next determined whether calcium-induced CaMKII signaling was required for melatonin-induced CREB phosphorylation. Calcium flux through calcium channels, a potent activator of CREB-dependent transcription, activated CREB via phosphorylation of serine 133. Calcium and EGTA, a Ca^2+^ chelator, did not change melatonin-enhanced p-CREB expression (Figure 3B,D). These results indicate that the melatonin-mediated CREB signaling pathway did not act through calcium channels. Furthermore, these results showed that CREB was phosphorylated by Raf–ERK–p90RSK–dependent signaling through the MT1 receptor, which was activated by melatonin. In addition, we confirmed whether each inhibitor of JNK and Akt associated with long-term memory processing blocked the expression of p-CREB. This result showed that melatonin did not change p-CREB expression through the JNK and Akt-related pathways (Figure 3E–H).

### 2.4. Melatonin Inhibited Senescence in ADR-Treated HT-22 Cells and Improved Memory Processing

**Figure 4:**
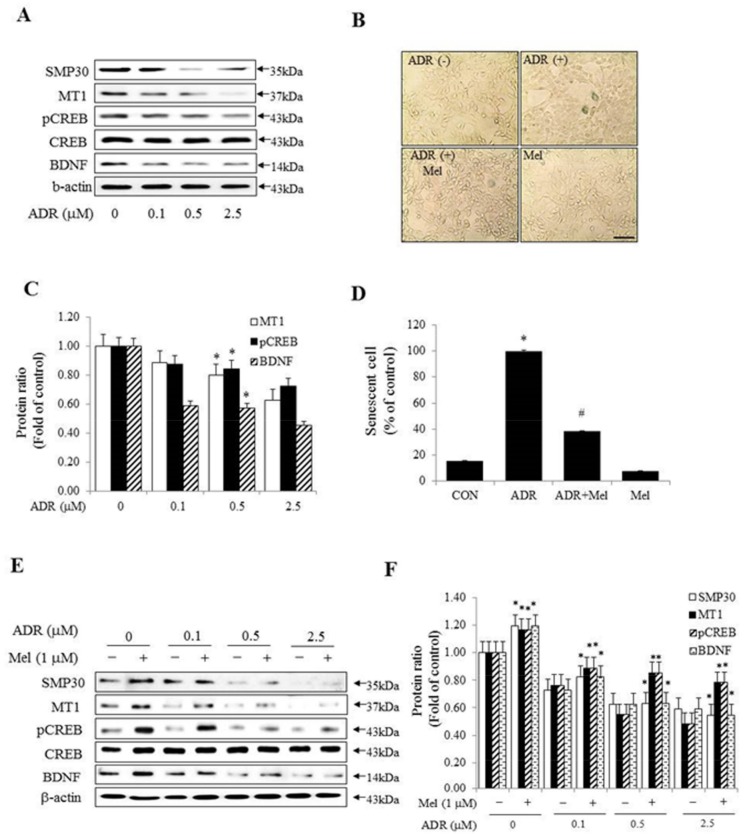
Melatonin inhibited senescence in ADR-treated HT-22 cells and improved memory processing. (**A**) HT-22 cells were treated with ADR (0, 0.1, 0.5, or 2.5 µM) for 4 h and the levels of SMP30, MT1, p-CREB, CREB, and BDNF were shown by Western blot analysis; (**B**) β-galactosidase (SA-β-gal) staining was performed for evaluation of the senescence status by treating with 0.5 µM ADR; (**C**) a graph representing the densitometry analysis of Figure 4A. * *p* < 0.05 versus each control group; (**D**) A graph representing the densitometry analysis of senescent cells. * *p* < 0.05 versus control group and # *p* < 0.05 versus ADR alone (scale bar = 100 µM); (**E**) The levels of SMP30, MT1, p-CREB, CREB, and BDNF were confirmed by Western blot analysis by treating with 0, 0.1, 0.5, and 2.5 µM ADR for 4 h and then stimulating with melatonin; (**F**) densitometry analyses for levels of these kinases are shown in Figure 4E. * *p* < 0.05 versus each absence of melatonin. All experimental values are given as means ± S.E.M (*n* = 3).

Next, some studies suggested that the pineal gland shows age-related changes and also, as it ages, the production of melatonin is reduced [2,38]. We investigated whether the expression of p-CREB and BDNF was reduced with in vitro cellular senescence. In the first instance, Western blot analyses of senescence marker protein-30 (SMP30), which is decreased in the cytosol during cellular senescence, revealed that adriamycin (ADR) gradually decreased SMP30 protein in the age-induced cell. The expression of MT1, p-CREB, and BDNF was diminished in an ADR-dose-dependent manner in HT-22 cells (Figure 4A,C). To investigate the possible role of melatonin-mediated memory in cellular senescence, we used a cell culture model of cellular senescence. The model involved the application of the anti-cancer drug ADR, which induces cellular senescence and quantification of senescence-associated β-galactosidase (SA-β-gal) cells and activity. ADR was applied to HT-22 cells either alone or in combination with melatonin. In SA-β-gal stain analysis, ADR treatment revealed an increase of about 80% in SA-β-gal positive cells compared with the control, while co-treatment of melatonin and ADR showed about a 60% decrease of the SA-β-gal positive cells compared with ADR treatment (Figure 4B,D). The Western blot assay also showed that the expressions of MT1, p-CREB, and BDNF were significantly higher in melatonin-treated cells than in untreated cells, in an ADR-dose-dependent manner (Figure 4E,F). These results suggested that there were differences in the expressions of p-CREB and BDNF according to the presence of melatonin with cellular senescence. 

## 3. Discussion

In this study, we demonstrated that melatonin mediated MT1–ERK–p90RSK–CREB–BDNF members of the long-term memory pathway in HT-22 cells. The hippocampus is a crucial organ in memory, but hippocampal malfunctions contribute to the pathogenesis of the disease, characterized by the progressive loss of memory in the elderly [39]. Thus, studies using hippocampal cellular models are important to understand molecular and cellular processes in the pathogenesis disease. Among the established hippocampal cell lines, H19-7 cells have been used for studies on the development and plasticity of hippocampal neurons [40]. On the other hand, HT-22 cells are known to be able to mimic long-term potentiation (LTP) and memory [41]. Based on this study, we first performed experiments with these two cell lines. Our results demonstrated that the MT1 receptor was significantly increased after melatonin treatment in HT-22 cells, whereas the MT2 receptor was not detected. On the other hand, in H19-7 cells, both p-CREB and BDNF were expressed at very low levels and the MT1 receptor was not expressed in the melatonin treatment. A possible explanation is that the distribution and expression of these receptors are tissue- and organ-dependent in mammals [42,43,44]. The MT1 receptor is located in the cerebellum, occipital, parietal, frontal and temporal cortex, thalamus, and hippocampus of rodents [45] and humans [9]. Conversely, the MT2 receptor is located in the rodent suprachiasmatic nucleus (SCN) [46]. These results suggest that the differences in expression of melatonin receptors are due to tissue and organ specificity.

Long-term memory formation requires gene expression and protein synthesis-dependent stabilization processes that take place in the brain, particularly in the hippocampus [47]. Among these genes and proteins, CREB and BDNF are induced in the processing of long-term memory and are sufficient for long-term memory processing in the hippocampus [48,49]. Here, we investigate the mechanisms for CREB-BDNF induced by melatonin in vitro in long-term memory processing, because the mechanism of increased CREB and BDNF by melatonin has not been elucidated yet. Long-term memory processing controls four signaling pathways: (i) calcium–calmodulin kinases and Ca^2+^ [50]; (ii) mitogen-activated protein kinase (MAPK) [51]; (iii) c-Jun N-terminal kinases (JNK) [52]; (iv) cAMP-dependent protein kinase (protein kinase A) and Akt [53,54]. All four pathways are combined into a signal to the CREB, which plays an important role in synaptic plasticity and long-term memory processing [55]. In the hippocampus, CREB is phosphorylated by PKA, PKC, and Ca^2+^/calmodulin-dependent kinases (CaMK) II following Ca^2+^ influx [56,57]. Additionally, many studies suggested that the modulation of diverse protein kinases directly or indirectly converges to activate CREB, mainly through PKA, CaMK, Ras/MAPK, and Akt [58,59,60,61,62,63,64]. Melatonin also plays an important role as a circadian modulator in memory processing [65]. Recently, Yoo et al. found that melatonin activated CREB in the dentate gyrus (DG) region of the hippocampus in aged mice [20]. Although the mechanisms of its memory-processing effects remain unclear, melatonin acts by binding MT1 and MT2 receptors in the hippocampus [11], and the MT1 receptor contributed to the regulation of p-CREB in the SCN neurons [66]. Also, melatonin can enhance memory via the activation of CREB, but the signaling mechanism between melatonin receptors and p-CREB has been unknown. Therefore, we used melatonin inhibitors and the signal members mentioned above to know the specificity of the Raf–ERK pathway by melatonin. This result suggested that the other three signals besides the Raf-ERK pathway are not involved in long-term memory processing by melatonin in vitro.

ERK activity has been suggested to be important for synaptic plasticity processes and memory formation and its inhibition leads to deficits in long-term synaptic plasticity and memory [67,68]. Thus, we investigated upstream and downstream mediators of ERK cascades involved in p-CREB activation. In the present study, ERK inhibitor specifically blocked p-p90RSK, p-CREB, and BDNF, which belong to ERK downstream, but did not inhibit phosphorylation of Raf and MT1 expression, which are upstream of ERK after melatonin treatment. Interestingly, among the intracellular targets of melatonin, the calcium/calmodulin complex plays a major role in both melatonin receptor-dependent and -independent functions. Activation of CaMKII also plays a central role in synapse formation, neuroplasticity, learning, and memory in the hippocampus [69,70]. Therefore, we studied whether calcium or CaMKII signals required melatonin-induced p-CREB activation. Figure 3B,D showed that both calcium and EGTA Ca^2+^ chelation did not affect melatonin-enhanced p-CREB. These results demonstrated that the melatonin-mediated CREB signaling pathway did not act through calcium channels. JNK is also involved in regulating synaptic plasticity and modulating long-term memory processing in hippocampus [52]. Thus, we examined whether a JNK inhibitor (SP600125) blocked the expression of p-CREB after melatonin treatment in this study. The JNK inhibitor did not cause any change in the expression of p-CREB by melatonin. Finally, Akt contributes to mechanisms associated with synaptic plasticity and long-term memory processing [53]. We also examined whether the Akt inhibitor (LY294002) blocked the expression of p-CREB by melatonin. The Akt inhibitor did not cause any change in the expression of p-CREB, as expected.

There have been several reports on the role of long-term memory processing in relation to p-CREB and BDNF for melatonin function in the pineal gland [71,72], and it is known that the pineal gland and melatonin are involved in aging and age-related diseases [3,15]. Melatonin declines significantly in middle age [73], and this decrease was hypothesized to be related to various age-related physiological changes including memory impairment [74,75]. This study showed that p-CREB and BDNF gradually increased with dose-dependent concentration of melatonin in HT-22 cells, which indicates that the decrease in melatonin as a result of the aging of the pineal gland leads to a decrease in the expression of p-CREB and BDNF in in vitro cellular senescence. We hypothesized that the dose-dependency of ADR treatment to HT-22 cells implies the senescence of hippocampal cells in vitro; as hippocampal cells age, the levels of p-CREB and BDNF expression in the hippocampus are reduced.

Melatonin increased CREB signaling members associated with long-term memory processing in HT-22 cells. Cheng and colleagues showed the differential effects of melatonin on hippocampal neurodegeneration in accelerated senescence prone mouse-8 (SAMP8) by initiating melatonin treatment at different ages in vivo [18]. Melatonin administration improved memory performance and attenuated the effect of aging on cognitive processing in vivo [76,77]. We investigated how the levels of the p-CREB and BDNF expression were altered by the effect of melatonin in the dose-dependent manner of ADR in vitro, similar to in vivo studies. Co-treatment with melatonin and ADR markedly reduced the number of SA-β-gal positive cells compared to ADR treatment alone [78], and the levels of p-CREB and BDNF expression decreased in a dose-dependent manner that corresponded to the ADR concentrations. However, those suppression effects of ADR were ameliorated by melatonin treatment. These results suggested that the melatonin reverse–dose–dependent treatment and ADR-dose-dependent treatment have the effect of reducing pineal gland production and hippocampus function, respectively, with age in vitro.

**Figure 5:**
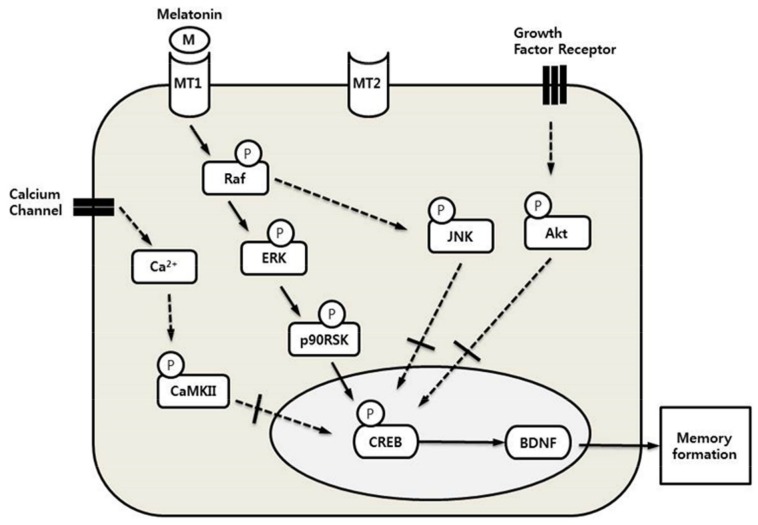
The proposed mechanism demonstrates a diagram of the CREB signaling pathway stimulated by melatonin in HT-22 cells. The effect of melatonin shows Raf-ERK-CREB cascades through the MT1 receptor. Long-term memory formation in the hippocampus is a highly dynamic process through this pathway.

In conclusion, these results showed that melatonin mediated the MT1-ERK–p90RSK–CREB–BDNF signaling pathway of long-term memory processing in vitro (Figure 5). Our study also demonstrated that melatonin significantly increased the levels of p-CREB and BDNF expression in HT-22 cells treated with melatonin. We suggest that the suppression of p-CREB and BDNF expression by cellular senescence is also improved by melatonin. The understanding of the melatonin–CREB signaling pathway associated with memory processing may stimulate therapeutic strategies for age-related cognitive deficits and neurodegenerative disorders.

## 4. Materials and Methods

### 4.1. Materials

Adriamycin (ADR) was supplied by Ildong Pharmaceuticals Co., Ltd. (Seoul, Korea) and dissolved in DMSO and water. Melatonin and calcium were purchased from Sigma (St. Louis, MO, USA). Antibodies recognizing p-p90RSK (RSK1/RSK2/RSK3; 32D7), p-CREB (Ser133), and CREB (48H2) were obtained from Cell Signaling Technology, Inc. (Beverly, MA, USA). SMP30 (K-18), PKAα cat (A-2), p-Akt1/2/3 (Ser 473), Akt1 (B-1), p-CaMKII (22B1), CaMKII (M-176), p-Raf (Ser 259), Raf (C-12), p-JNK (G-7), JNK (D-2), p-ERK1/2 (Thr 202/Tyr 204), ERK1/2 (MK1), BDNF (N-20), MEL-1A-R (R-18) and MEL-1B-R (T-18) were purchased from Santa Cruz Biotechnology (Santa Cruz, CA, USA). Melatonin-1A-R siRNA (m) and con siRNA-A were also purchased from Santa Cruz Biotechnology. Melatonin-1A-R siRNA (m) is used for the inhibition of MEL-1A-receptor expression in mouse cells. A monoclonal anti-β-actin antibody was purchased from Sigma-Aldrich (Darmstadt, Germany). Luzindole (L2407), a melatonin receptor antagonist, was provided by Sigma-Aldrich and dissolved in DMSO. The β-galactosidase staining kit was purchased from Mirus (Madison, WI, USA). The Fluor™ 405- and 488-labeled secondary antibodies were obtained from AnaSpec, Inc. (Fremont, CA, USA) for immunofluorescent analysis. The ERK inhibitor II (FR180204, SC-203945), a novel ERK-selective inhibitor, was purchased from Santa Cruz Biotechnology. JNK inhibitor (SP600125, S5567) and Akt inhibitor (LY294002, A6730) were purchased from Sigma-Aldrich and dissolved in DMSO. EGTA (E3889) was purchased from Sigma-Aldrich. Three melatonin agonists were purchased as follows. Ramelteon (R016) was purchased from Sigma. 2-iodomelatonin and ramelteon metabolite M-II (SC-203463 and SC-219935, respectively) were obtained from Santa Cruz Biotechnology.

### 4.2. Cell Culture

HT-22 cells, an immortalized mouse hippocampal cell line, were a generous gift from Dr. Sang-Hyun Kim (Kyungpook National University, Daegu, Korea). H19-7 cells were derived from hippocampi dissected from Holtzman rats and purchased from ATCC (No. CRL-2526, Manassas, USA). HT-22 cells and H19-7 cells were cultured in DMEM with 2 mM glutamine, 100 units/mL penicillin, and 100 μg/mL streptomycin and supplemented with 10% FBS (GIBCO, Grand Island, NY, USA) in a CO_2_ incubator (5% CO_2_/95% air, 37 °C). Cells were sub-cultured once every three days. Four hours after treatment with 500 nM ADR, β-galactosidase (SA-β-gal) staining was performed to evaluate the senescent status of the hippocampal cells.

### 4.3. Senescence-Associated β-Galactosidase (SA-β-gal) Staining

HT-22 cells were seeded in 35-mm culture dishes and treated with ADR for 4 h and melatonin for 2 h. The cell culture medium was aspirated and cells were washed with PBS. The cells were fixed with 4% formaldehyde for 3 min at room temperature and stained with β-galactosidase.

### 4.4. Western Blot

Whole cell extracts were prepared using RIPA buffer. The protein concentration was quantified with a protein assay reagent from Bio-Rad (Hercules, CA, USA). Equal amounts of protein (30 μg) were mixed with Laemmli Sample Buffer (Bio-Rad) and heated for 5 min at 100 °C prior to loading. Total protein samples were subjected to 10% SDS–polyacrylamide gel electrophoresis (SDS-PAGE) and electrophoretically transferred onto a PVDF membrane. The membranes were blocked with 5% non-fat milk, incubated with primary antibodies at a dilution of 1:1000 overnight at 4 °C in PBS-T, and washed with three changes of wash buffer. The membranes were incubated with a second antibody and exposed using ECL reagents.

### 4.5. Transfection of siRNA

HT-22 cells were transfected with siRNA using the Lipofectamine 2000 reagent according to the manufacturer’s instructions. In brief, aliquots of 1 × 10^4^ cells were plated onto six-well plates and grown to approximately 70% confluence. The cells were then transfected with control siRNA and melatonin-related receptor siRNA plus 100 pmol of Lipofectamine for 6 h in Opti-MEM^®^ I (Invitrogen, Waltham, MA, USA). Following an incubation period of 48 h, various protein levels were quantified using Western blot analysis.

### 4.6. Immunofluorescence

For immunochemistry, HT-22 cells were seeded on coverslips in plates, ADR treated for 4 h, and then treated with melatonin and luzindole, each for 2 h. The cells were fixed in 4% formaldehyde, permeabilized with 0.5% Triton X-100 in PBS, and blocked with 10% normal goat serum in 0.5% Triton X-100. The p-CREB primary antibody (Cell Signaling Technology, Inc.) was used at 1:100 and incubated with cells overnight at 4 °C. Following three PBS washes, the cells were incubated with AlexaFluor™ 405- or 488-labeled secondary antibodies diluted to 1:250 for 1 h at room temperature. After a final PBS wash, the slides were mounted with ProLong Gold Anti-Fade reagent onto glass slides. Immunofluorescent images were taken using a Zeiss confocal microscope (Carl Zeiss AG, Oberkochen, Germany).

### 4.7. Statistical Analysis

All data are represented as the mean ± S.E.M. Differences between datasets were assessed by analysis of variance (ANOVA) followed by Bonferroni post hoc analysis. A value of *p* < 0.05 was considered statistically significant.
